# Biosynthesis of Isoprene Units in *Euphorbia lathyris* Laticifers vs. Other Tissues: MVA and MEP Pathways, Compartmentation and Putative Endophytic Fungi Contribution

**DOI:** 10.3390/molecules24234322

**Published:** 2019-11-26

**Authors:** Clément Gastaldo, Agata Lipko, Estelle Motsch, Pierre Adam, Philippe Schaeffer, Michel Rohmer

**Affiliations:** 1 Institut de Chimie, Université de Strasbourg/CNRS UMR 7177, Institut Le Bel, 4 rue Blaise Pascal, 67070 Strasbourg CEDEX, France; gastaldo@outlook.fr; 2 Institute of Biochemistry and Biophysics, Polish Academy of Sciences, Pawinskiego 5A, 02-106 Warsaw, Poland; ag.lipko@gmail.com; 3 Biogéochimie Moléculaire, Institut de Chimie, Université de Strasbourg/CNRS UMR 7177, Institut Le Bel, 4 rue Blaise Pascal, 67070 Strasbourg CEDEX, France; emotsch@unistra.fr (E.M.); padam@unistra.fr (P.A.); p.schaef@unistra.fr (P.S.)

**Keywords:** *Euphorbia lathyris*, *Euphorbiaceae*, isoprene unit biosynthesis, isoprenoids, isotope ratio GC-MS, latex, MEP pathway, MVA pathway, triterpenes

## Abstract

*Euphorbia* species are characterized by a net of laticifers producing large amounts of triterpenes. These hydrocarbon-like metabolites can be converted into fuel by the methods of the oil industry. *Euphorbia lathyris* is easily grown at an industrial scale. In an attempt to increase its triterpene production, the metabolic pathways leading to isoprenoid were investigated by incorporation of ^13^C labeled glucose and mevalonate and ^2^H labeled deoxyxylulose as well as by natural abundance isotope ratio GC-MS. Latex triterpenes are exclusively synthesized via the mevalonate (MVA) pathway: this may orient future search for improving the triterpene production in *E. lathyris*. Phytosterols and their precursors are mainly derived from MVA pathway with a slight contribution of the methylerythritol phosphate (MEP) pathway, whereas phytol is issued from MEP pathway with a minor contribution of the MVA pathway: this is in accordance with the metabolic cross-talk between cytosolic and plastidial compartments in plants. In addition, hopenol B behaved differently from the other latex triterpenes. Its ^13^C isotope abundance after incorporation of ^13^C labeled glucose and its natural abundance δ^2^H signature clearly differed from those of the other latex triterpenes indicating another metabolic origin and suggesting that it may be synthesized by an endophytic fungus.

## 1. Introduction

**Figure 1:**
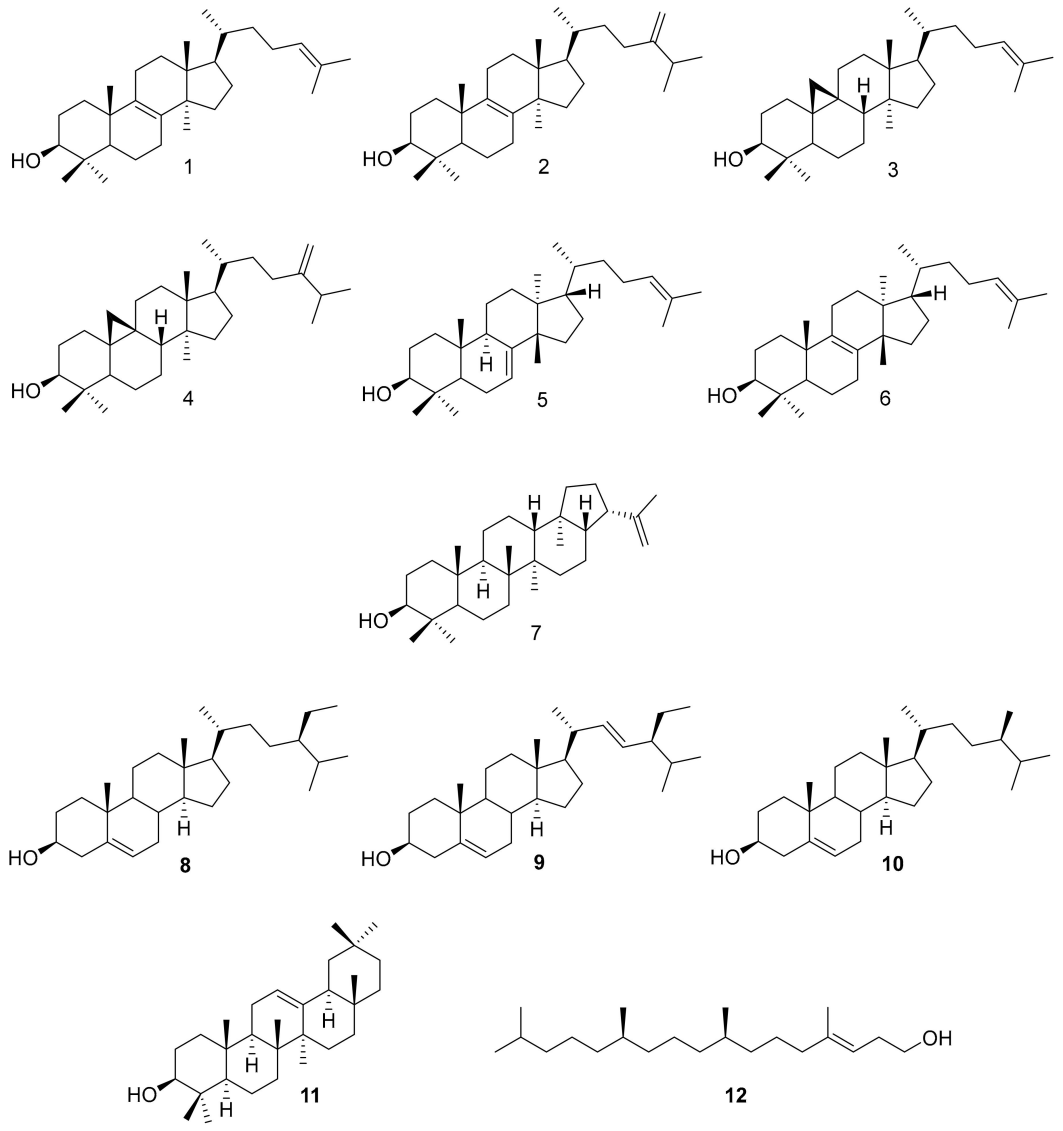
*E. lathyris* isoprenoids: **1**, lanosterol; **2**, 24-methylene lanostenol, **3**, cycloartenol; **4**, 24-methylene cycloartanol; **5**, butyrospermol; **6**, euphol; **7**, hopenol B; **8**, sitosterol; **9**. stigmasterol; **10**, campesterol; **11**, β-amyrin; **12**, phytol.

Among the *Euphorbiaceae*, the genus *Euphorbia* includes ca. 2000 species with no taxonomic subdivision although the existence of four clades has been proposed [1]. They are now accepted as subgenera [2,3]. All species are characterized by a net of non-articulated laticifers. These specialized cells represent a closed system devoted to the production and storage of latex, a white, milky liquid [4]. This latex is characterized by a high content of isoprenoids, mainly triterpenes with a rather large structural diversity [5,6], and to a lesser extent of diterpenoids, most of them being irritant toxics, causing intense inflammatory reactions and acting often as procarcinogens [7,8]. The triterpene fingerprint of *Euphorbia* latex has been used as chemotaxonomic marker and allowed division of the huge genus *Euphorbia* into subgroups according to the triterpene composition of the latex [5,6]. Plant triterpenes are derived from enzymatic cyclization of oxidosqualene and are characterized by a C_30_H_50_O crude chemical formula [9,10,11], which makes them suitable for fuel production by the methods used by the oil industry [12,13]. In the frame of the European EULAFUEL project, it was attempted to improve the triterpene content of the latex of *Euphorbia lathyris* (Figure 1). *E. lathyris* is originally a Mediterranean plant, which is now subcosmopolite. It can be easily grown, even on poor soils, with yields compatible with fuel production [14,15].

**Scheme 1:**
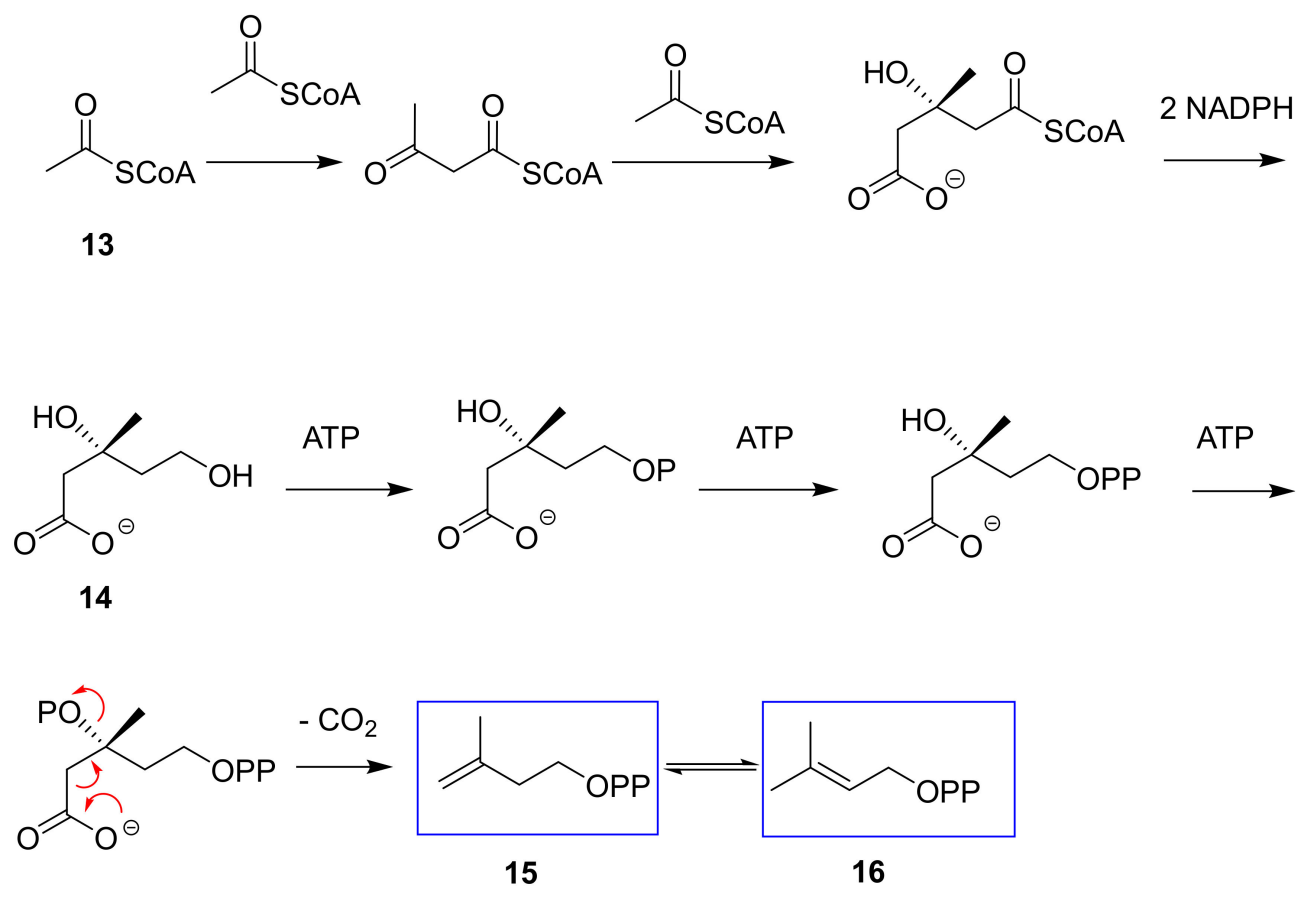
Mevalonate pathway for the biosynthesis of isoprene units: **13**, acetyl-CoA; **14**, mevalonate (MVA); **15**, isopentenyl diphosphate (IPP); **16**, dimethylallyl diphosphate.

**Scheme 2:**
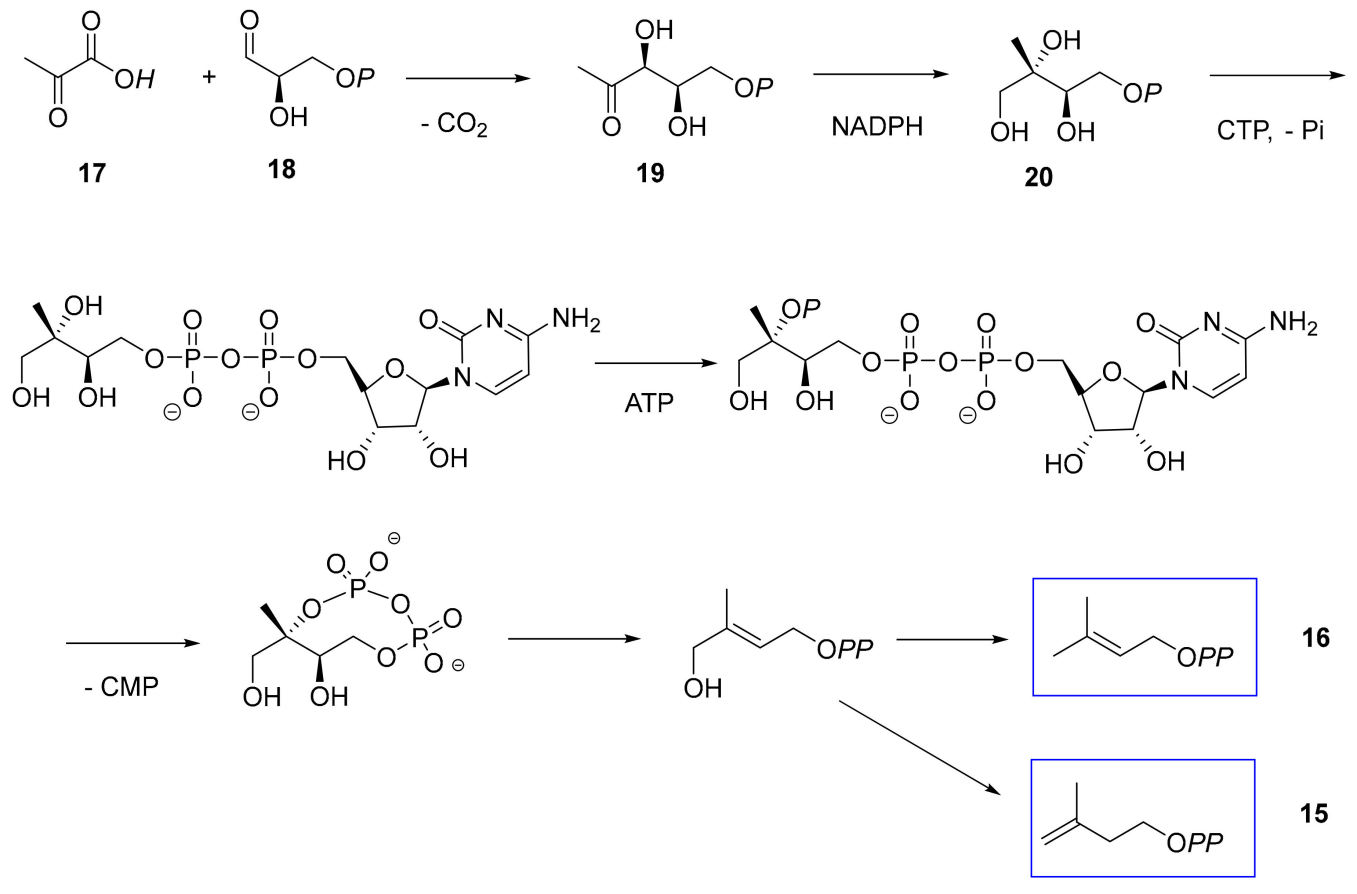
Methylerythritol phosphate pathway for the biosynthesis of isoprene units: **15**, IPP; **16**, DMAPP; **17**, pyruvate; **18**, d-glyceraldehyde 3-phosphate; **19**, 1-deoxy-d-xylulose 5-phosphate; **20**, 2*C*-methyl-d-erythritol 4-phosphate. (MEP).

Two pathways are operating in plants for the biosynthesis of isopentenyl diphosphate (**15**, IPP, Scheme 1 and Scheme 2) and dimethylallyl diphosphate (**16**, DMAPP, Scheme 1 and Scheme 2), the universal precursors of isoprene units: the long known mevalonate (MVA) pathway found in the cytosol (Scheme 1) [16] and the methylerythritol phosphate (MEP) pathway, first found in bacteria [17,18], and localized in plants only in plastids (Scheme 2) [19,20,21,22]. 

This dichotomy is roughly respected. Incorporation experiments performed with precursors labeled with stable isotopes (mainly ^13^C) have shown that triterpenes including sterols, which are all derived from oxidosqualene, most sesquiterpenes and the side-chain of ubiquinone are derived from the MVA pathway whereas hemi-, mono- and diterpenes, carotenoids and the side chain of plastoquinone synthesized in the plastids are issued from the MEP pathway [21,22]. Cross-talk between the two cellular compartments has, however, often been observed, resulting in terpenoids with isoprene units of mixed origins [19,20,21,22,23]. In order to enhance the triterpene content of the *E. lathyris* latex by genetic modification, it was capital to know the biosynthetic pathway(s) leading to them in order to be capable of manipulating them.

**Scheme 3:**
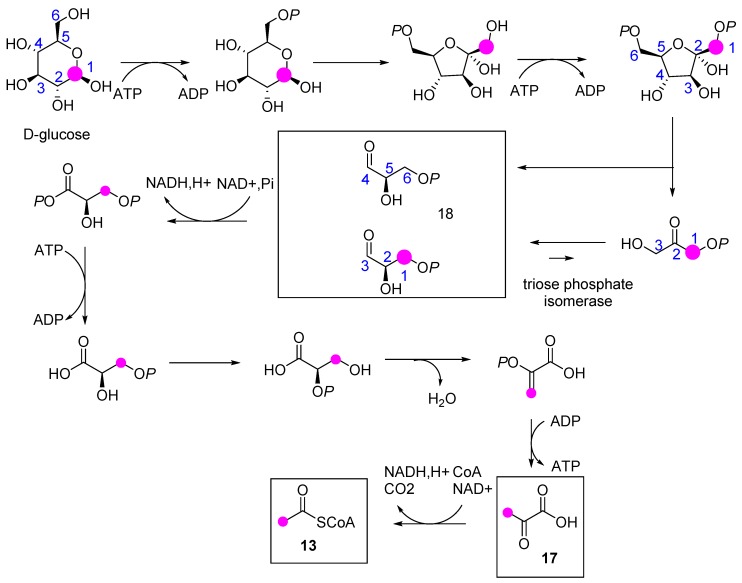
Catabolism of (1-^13^C)glucose via glycolysis and triose phosphate pathway towards d-glyceraldehyde 3-phosphate **18**, pyruvate **17** and acetyl-CoA **13**, the precursors of the MEP and MVA pathways.

**Scheme 4:**
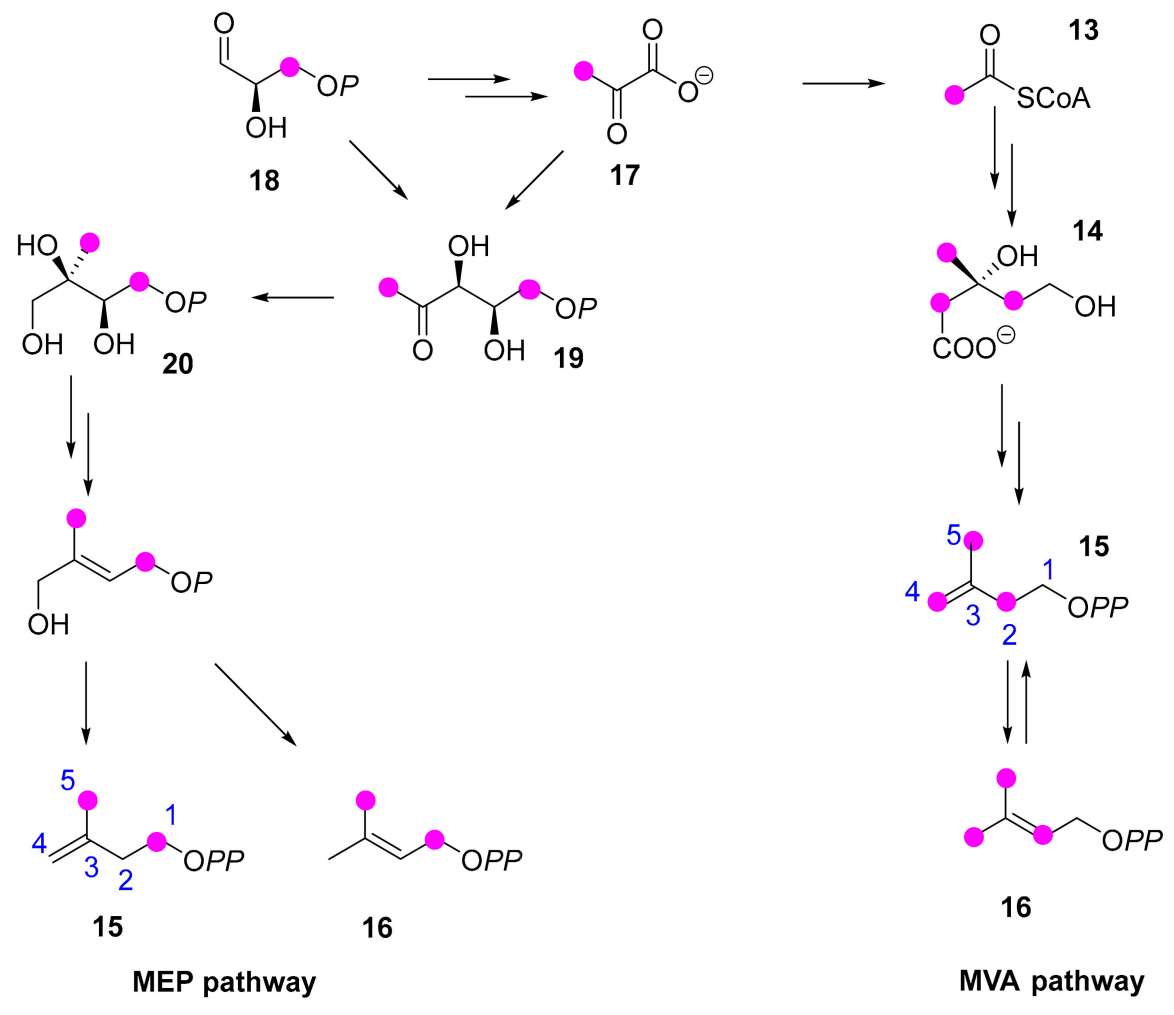
Incorporation of (1-^13^C)glucose into IPP **15** and DMAPP **16** via the MVA or the MLEP pathway.

Biosynthesis of triterpenes in *Euphorbia* latex was investigated a long time ago using radioactive ^14^C-labeled mevalonate, which was incorporated into the triterpenes [24,25,26]. The method is quite sensitive, but does not allow to detect the possibility of an alternative pathway. In addition, the radiochemical characterization methods are based on the identity of a natural abundance marker corresponding to the analyzed radiolabeled metabolite and introduced in the mixture to be compared by physical and chemical methods (chromatography, recrystallization to constant specific activity, preparation of derivatives) with the putative radiolabeled metabolite. Results are thus always hampered by the possibility that the marker may not be differentiated from an unknown radiolabeled structurally different metabolite [27]. For our purpose, we switched to labeling experiments using mainly ^13^C-labeled glucose, which is often well accepted by plants and a universal precursor for all metabolite that allows differentiating MVA and MEP pathways by the resulting labeling pattern in isoprene units (Scheme 3 and Scheme 4) [18,21]. ^13^C-labeled MVA, which is the specific precursor of the MVA pathway or ^2^H-labeled deoxyxylulose as specific precursor of the MEP pathway [28] may be utilized to complete the ^13^C glucose incorporation experiments. Detection of the labeling was done by NMR spectroscopy for the ^13^C after glucose or MVA labeling when the isotope abundance was high enough. This method allows unambiguous localization of the labeled positions and thus of the metabolic pathway(s) leading to this labeling pattern. The study was completed by isotope ratio GC-MS (IR GC-MS) analyses after incorporation of ^2^H-labeled deoxyxylulose when the isotope abundance was very low.

Labeling experiments brought an interesting harvest of data, some being rather unexpected. *E. lathyris* latex triterpenes are solely derived from MVA pathway. There is a strong compartmentation inside the plant: with the exception of glucose, the other precursors (deoxyxylulose, MVA) do not reach the laticifers. Cross-talk between MVA and MEP pathways was observed for the terpenoids, which are present outside the laticifers. The labeling pattern and the ^13^C and ^2^H natural abundance isotope signature was different for the latex triterpene hopenol B and the other latex triterpenes (all derived from MVA pathway), indicating a different metabolic origin and suggesting that hopenol B might be a fungal endophyte metabolite.

## 2. Results

### 2.1. Latex Triterpenes and Other Isoprenoids in E. lathyris

#### 2.1.1. Plant Material and Incorporation Conditions for Labeled Precursors

Labeling of metabolites using precursor labeled with a stable isotope (^13^C or ^2^H) and detection of the label by NMR spectroscopy requires the incorporation of rather large amounts of precursor. This task is usually rather difficult to perform. *E. lathyris* is a bisannual herbaceous plant, and several attempts were made to find out the best experimental conditions: either adult plants in their second year just before flowering grown either outdoor in open ground in the garden of one of us (MR) or indoor in large pots or axenic plantlets obtained from seeds thoroughly surface sterilized and grown for three weeks in sterile conditions.

*E. lathyris* plantlets grown in sterile conditions were obtained from seeds whose surface was carefully sterilized. Due to dormancy problems, germination of *E. lathyris* seeds is rather erratic and poorly reproducible. Germination could be improved by treating the seeds with gibberellic acid GA3. Surface sterilized seeds were thus maintained in sterile conditions in Petri dishes at 28 °C in the dark on filter paper soaked with a water solution of GA3 (1 mM, optimal concentration corresponding to ca. 87% germination yield) [29,30]. After germination, when the rootlets were ca. 1–2 cm long, the plantlets were transferred into graduated 2 L glass cylinders on a sterile solid substrate based on mineral Murashige-Skoog (MS) medium [31] containing the labeled precursor and left on an East oriented window sill at room temperature. After ca. 3 weeks growth, the plantlets (ca. 20–35, corresponding to 10–25 g, fresh weight) were harvested and lyophilized. Agarose medium proved to be the best solution for rooting vs. vermiculite or rock wool.

#### 2.1.2. *E. lathyris* Isoprenoids

All latex triterpenes, sitosterol (by far the major phytosterol) and phytol were extracted from freeze-dried plant material and isolated by preparative thin layer chromatography (TLC) and isolated after acetylation as acetate derivatives, which were further purified by TLC on silica gel or on AgNO_3_ impregnated silica gel when this was required. Identification was made by GC, GC-MS, ^1^H- and ^13^C-NMR spectroscopy and comparison of the data with those of authentic reference material.

In order to determine the best plant material and the most suitable labeling conditions for outdoor grown adult plants, latex from such plants, plants grown indoor in pots and plantlets grown in axenic conditions were separately analyzed. Adult outdoor grown plants contained the highest triterpene concentration, the triterpenes of isolated latex representing 20% of the mass of the dry latex, suggesting that the size and the volume of the laticifers were enhanced as compared to those of other plant tissues. Younger plants and plantlets contained, however, enough material to perform labeling experiments (Table 1). Although the presence of laticifers was reported in roots from several *Euphorbia* species [32], they are of smaller size than those of aerial parts and are absent in roots of diameter lower than 1–2 mm [4]. It has been shown in addition that roots do not represent a major site for triterpene biosynthesis in these plants [33]. Accordingly, roots were not investigated in the labeling experiments. The major latex triterpenes are lanosterol (**1**), cycloartenol (**3**), 24-methylene cycloartanol (**4**), butyrospermol (**5**) and hopenol B (**7**; Figure 1). Some minor triterpenes representing less than 5% of the total triterpenes were also found: euphol (**6**) and 24-methylene lanostenol (**2**) from the latex and β-amyrin (**11**) from other plant tissues. Relative amounts are quite similar in the isolated latex or in whole plants, independently of their age and their size (Table 2) and correspond to those previously reported in the literature [5,6,15,34,35]. 

Interestingly, the triterpene profile is similar in a whole plant, in isolated latex and in a plant whose latex has been partially removed by cutting, indicating that even in the case of an anastomosed laticifer system, the totality of the latex cannot be collected. This justified the fact that only whole plants were analyzed in labeling experiments and not only the latex.

Sitosterol (**8**, 82% of total sterols, Figure 1) was accompanied by small amounts of stigmasterol (**9**, 10 %) and campesterol (**10**, 5%) and was utilized as it is without further separation. Most carbon atoms of the three steryl acetates (with the exception of those of the side-chain) are characterized by identical chemical shifts in ^13^C-NMR spectra. Relative amounts did not vary from one analysis to another, and the phytosterol concentration (*w/w*, freeze-dried plant material) was constant as expected for an essential component of the plasma membrane.

### 2.2. E. lathyris Labeling with (1-^13^C)Glucose

Adult plants (grown outdoor in the open ground or indoor in a pot) were cut just above two opposite axillary buds near the bottom of the stalk. (1-^13^C)Glucose (0.5 g in 0.5 mL water, isotope abundance 99%) was introduced with a syringe in the medullary part of the stalk at the level of the cutting. This process was repeated once again after 3 weeks. After 4 additional weeks, the plants were harvested after the axillary buds developed into ca. 5 cm long branchlets, freeze-dried and extracted.

Alternatively, plantlets were grown in axenic conditions in the presence of (1-^13^C)glucose added to the solid MS medium (1%, 55 mM, isotope abundance 99%) during 3 weeks. Results were qualitatively identical in all experiments. (1-^13^C)Glucose is metabolized vie glycolysis and the triose phosphate pathway yielding glyceraldehyde 3-phosphate (**18**) and pyruvate (**17**), the precursors of the MEP pathway (Scheme 3 and Scheme 4). Pyruvate (**17**) is further converted into acetylcoenzyme A (**13**), the precursor of the MVA pathway (Scheme 3 and Scheme 4). The same carbon atoms were labeled in all isoprene units in all analyzed isoprenoids (Table 3 and Table 4). ^13^C isotope abundances were lower in the isoprene units of adult plants (very low in those of the outdoor grown plant, close to the detection limit) than in those of the plantlets. In latex triterpenes (**1**–**7**) as well as in sitosterol (**8**), only carbon atoms corresponding to C-2, C-4 and C-5 are labeled as expected from the MVA pathway (Table 3 and Table 4, Scheme 4) [18,21]. 

In contrast, for phytol (**12**), only C-1 and C-5 carbon atoms derived from IPP are labeled according to the MEP pathway as expected for a diterpene (Table 3 and Table 4, Scheme 4) [18,21]. In the plantlet isoprenoids, the carbon atoms derived from C-1 and C-3 of IPP are slightly (but significantly) labeled in the isoprene units of the latex triterpenes. As C-3 is never labeled in the MEP pathway, this labeling does not arise from a contribution of the MEP pathway. Glucose is involved in many metabolic pathways and is converted partly into CO_2_. This means that ^13^CO_2_ is produced from (1-^13^C)glucose and recycled by photosynthesis in all plant metabolites, resulting in some scrambling and in a uniform ^13^C-enriched background. A similar conclusion is drawn from labeling pattern of phytol (**12**) where C-2, C-3 and C-4 carbon atoms derived from IPP should not be labeled from the MEP pathway. Interestingly, this background is higher in phytol (**12**) than in latex triterpenes and sterols (**8**-**10**). Phytol (**12**) is synthesized in chloroplasts, the site where ^13^CO_2_ from catabolism is recycled by photosynthesis.

The incorporation of (1-^13^C)glucose of 99% isotope abundance results only in a modest isotope abundance around 35% in isoprenoids. This means that the labeled precursor is not the main carbon source for these compounds. The plants use essentially natural abundance atmospheric CO_2_ and/or the stocks of the cotyledons of the axenically grown plantlets. Previous studies indicated that *E. lathyris* plantlets use preferentially triacylglycerols from the endosperm rather than photosynthesis as carbon source [36,37]. The weight ratio between an *E. lathyris* freeze-dried plantlet and a seed (evaluated by weighing 30 lyophilized plantlets and 30 seeds) is close to 1, suggesting that most of the biomass of a plantlet may mostly arise from the seed reserves.

Contribution of the endosperm reserves is corroborated by the labeling pattern of fatty acids. In both adult plants grown on earth in a flowerpot and plantlets grown in axenic conditions on agar MS medium, only the even-numbered carbon atoms of linoleic acid are labeled (2.4% isotope abundance) according to glucose catabolism into acetyl CoA whereas none of the oleic carbon atoms is labeled (data not shown). Oleic acid is by far the most abundant fatty acid of the endosperm triacylglycerols. This suggests that only significant de novo biosynthesis of linoleic acid from (1-^13^C)glucose is observed whereas the natural ^13^C abundance oleic acid from the endosperm is utilized as it is. In addition, the methyl group of S-adenosylmethionine (SAM) is also labeled after incubation with (1-^13^C)glucose and is transferred onto the triterpene skeleton by methyltransferases. The methylene group of 24-methylene cycloartanol (**4**) and the methyl and ethyl groups of the phytosterol side-chains (**8**–**10**) are accordingly also ^13^C labeled. For the sake of clarity, only mean ^13^C isotope abundance are indicated.

### 2.3. E. lathyris Plantlet Labeling with (1,6-^13^C_2_)Glucose

Incorporation of doubly-labeled (1,6-^13^C_2_)glucose in the place of (1-^13^C)glucose should result in a higher isotope abundance of the labeled carbon atoms of isoprene units as C-1 and C-6 of glucose are metabolically equivalent (Scheme 3). Indeed glycolysis of (1,6-^13^C_2_)glucose affords glyceraldehyde 3-phosphate and dihydroxyacetone phosphate (Scheme 3), which are both ^13^C-labeled and interconvertible via the triose phosphate isomerase. Accordingly, the resulting pyruvate issued from triose phosphate metabolism and acetyl-CoA possess a double ^13^C isotope abundance as compared to that derived from singly labeled (1-^13^C)glucose. Experimental conditions were similar to those utilized for the (1-^13^C)glucose incorporation. Labeling patterns were identical with those obtained after incorporation of (1-^13^C)glucose with no enhancement of the isotope abundances as expected. Conclusions are identical: i) isoprene units of latex triterpenes **1**–**7** and sitosterol (**8**) are derived from MVA pathway; ii) those of phytol (**12**) from MEP pathway; the same slight scrambling occurred for all carbon atoms. Interestingly, hopenol B (**7**) showed a different behavior as compared to those of the other latex triterpenes: the isotope abundance of all labeled carbon atoms was always slightly but reproducibly higher than those of all other isoprenoids (Table 5).

### 2.4. E. lathyris Labeling with(3R,S)-(2-^13^C)Mevalonate

**Scheme 5:**
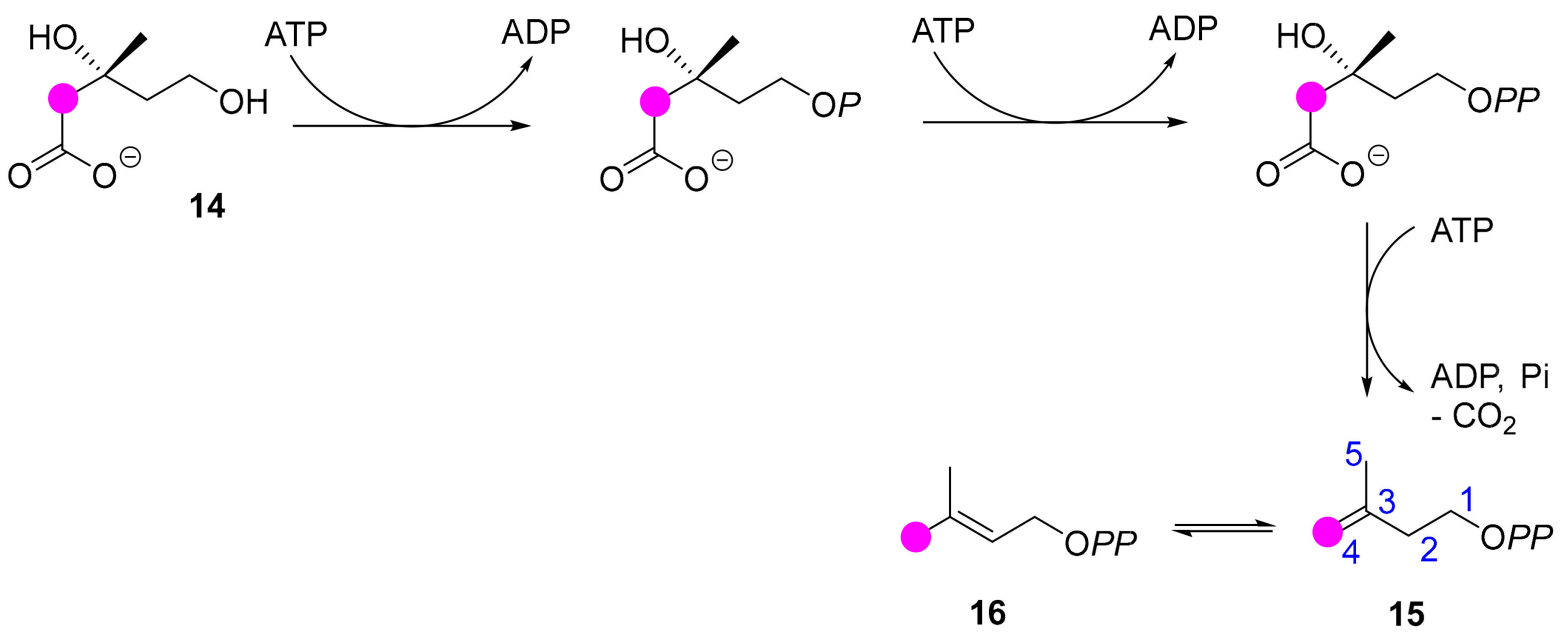
Incorporation of (3*R*,*S*)-(2-^13^C)MVA **14** into IPP **15** and DMAPP **16**.

Former assays using ^13^C-labeled glucose showed that latex triterpenes (**1**–**7**) and sitosterol (**8**) are synthesized via the MVA pathway. Incubation of (3*R*,*S*)-(2-^13^C)mevalonate should accordingly result in the labeling of their isoprene units (Scheme 5). Racemic (3*R*,*S*)-(2-^13^C)mevalonate is commercially available, but only the 3*R* enantiomer is a substrate of the mevalonate kinase and is incorporated into isoprenoids [38]. Commercial racemic (3*R*,*S*)-(2-^13^C)mevalonolactone (98% isotope abundance) was dissolved in water, and the pH of the solution was adjusted to pH 9 to convert the lactone into the mevalonate potassium salt, which is better incorporated than the lactone.

This stock solution was added to the MS medium (containing no carbon source) of *E. lathyris* plantlets grown in axenic conditions to reach a final 0.95 mM concentration of (3*R*)-(2-^13^C)mevalonate corresponding to concentrations recommended in the literature [23]. ^13^C labeling was found at the level of the carbon atoms derived from C-4 of IPP (corresponding to C-2 of mevalonate) in the isoprene units of cycloartenol (**3**), 24-methylene cycloartanol (**4**) and sitosterol (**8**) (Table 6). No ^13^C labeling was found in lanosterol (**1**), butyrospermol (**5**) and hopenol B (**7**). According to the experiments performed with ^13^C-labeled glucose, all isoprene units of these triterpenoids are synthesized via the MVA pathway. Qualitatively similar results were obtained with (3*R*,*S*)-(2-^13^C)mevalonolactone (0.4 mM), but with lower isotope abundance at the level of the labeled carbon atoms.

There is a clear dichotomy at the level of the latex triterpenoids. The three non-labeled triterpenes lanosterol (**1**), butyrospermol (**5**) and hopenol B (**7**) are only found in the latex. Cyclartenol (**3**) and 24-methylenecycloartanol (**4**) in contrast are present in large amounts in the latex, but are also synthesized in all plant cells as precursors for the phytosterols, which are essential constituents of the plasma membrane [10]. In addition, the latex triterpenes of the plantlets are only present in low concentration (0.1 mg·g^−1^, dry weight) in the seeds, indicating that most of these latex triterpenes do not derive from a pool already present in the seed but are synthesized de novo.

These results of the labeling experiments may be interpreted as follows. The exogenous mevalonate does not enter into the laticifers, but is utilized in the cytoplasm of the other cells for the biosynthesis of phytosterols, hence explaining the labeling of sitosterol (**8**) and of its two precursors cycloartenol (**3**) and 24-methylene cycloartanol (**4**). As sterol precursors, cycloartenol **3** and 24-methylene cycloartanol (**4**) are usually present in low concentrations with a high turn-over, resulting possibly in an intense labeling, which is diluted by the corresponding natural abundance isotopomers present in the latex.

The slight labeling found in phytol (**12**) (which is essentially synthesized via the MEP pathway according to the ^13^C labeled glucose incorporation experiments) on carbon atoms derived from C-4 of IPP corresponds to a minor contribution of the MVA pathway (Table 6). Such a cross-talk between MVA and MEP pathway is regularly found in plant systems [19,23].

### 2.5. E. lathyris Labeling with (5,5-^2^H_2_)Deoxyxylulose

1-Deoxy-d-xylulose 5-phosphate (DXP) is the first C_5_ precursor of the MEP pathway (Scheme 2). Bacteria and plant cell cultures incorporate, however, into isoprenoids the non-phosphorylated free 1-deoxy-d-xylulose (DX), which has to be phosphorylated by a non-specific d-xylulose kinase into DXP before entering the MEP pathway [39,40]. A possible contribution of the MEP pathway to the isoprenoid biosynthesis in *E. lathyris* was thus investigated using (5,5-^2^H_2_)deoxy-d-xylulose added to the culture medium of plantlets grown in axenic conditions (1 mM) [23,41]. GC-MS analysis of all isoprenoids revealed no measurable deuterium incorporation into phytol (**12**), the latex triterpenes **1**–**7** and sitosterol (**8**). The absence of labeling is most probably not due to the absence or the lack of activity of the d-xylulose kinase, as this enzyme is ubiquitous in plants [42]. An alternative would be a poor incorporation via the roots by *E. lathyris*.

**Scheme 6:**
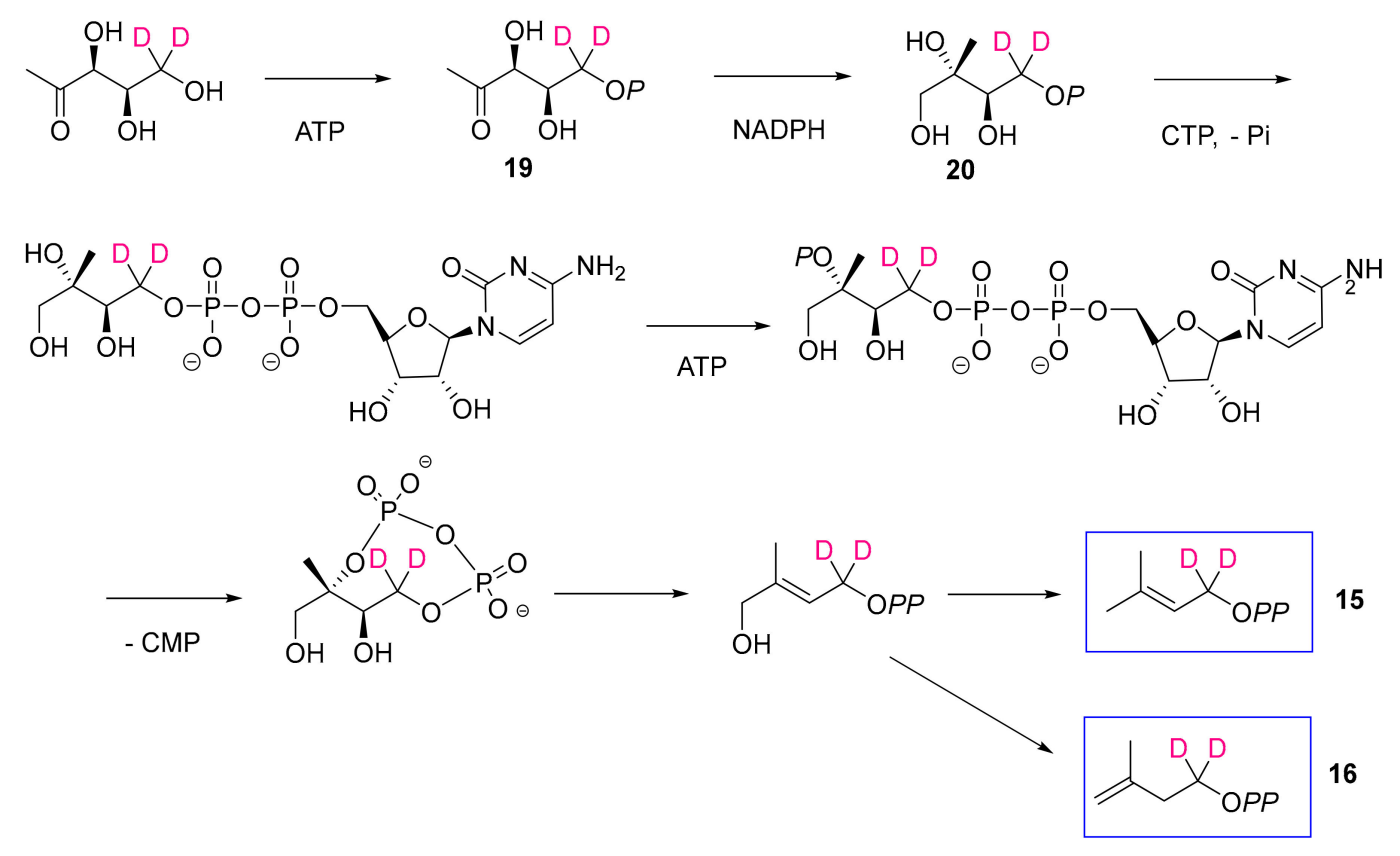
Incorporation of (5,5-^2^H_2_)deoxy-d-xylulose into IPP **15** and DMAPP **16**.

To check the possible deuterium incorporation in the isoprene units, gas chromatography-isotope ratio mass spectrometry (IR GC-MS), a quite sensitive method, was utilized, allowing determination of the ^2^H content by measuring the δ^2^H (‰) values of individual compounds corresponding to the deviation of the ^2^H/^1^H ratios relative to that of an universal reference sample [43,44]. This method for deuterium detection proved to be efficient in labeling experiments with a deuteriated precursor for the biosynthesis of bacterial triterpenes of the hopane series [45]. Results are consistent with those obtained after incorporation of (2-^13^C)MVA. The δ^2^H values were determined only once and are only indicative, but can be compared between them. Phytol (**12**) shows the highest δ^2^H isotope signature, which is consistent with a main origin via the MEP pathway. Sitosterol (**8**), mainly derived from the MVA pathway, is significantly labeled, indicating again a contribution of the MEP pathway to the biosynthesis of its isoprene units. For the latex triterpenes, the values have to be compared with those obtained with natural abundance material, where the δ^2^H values are comprised between −260 and −180‰ (see next Section 2.6). Lanosterol (**1**) and butyrospermol (**5**), which are only present in the latex, show a low isotope signature, close to those found in the triterpenes from natural abundance plantlets. Cycloartenol (**3**) and 24-methylene cycloartanol (**4**) are characterized by an intermediate isotope signature, much lower than that of sitosterol (**8**) but higher than those of the two former latex triterpenes. This suggests again that the values obtained for cycloartenol (**3**) and 24-methylene cycloartanol (**4**) correspond to those resulting from two different triterpene pools: one pool corresponding to the cycloartenol (**3**) and 24-methylene cycloartanol (**4**) from the latex, which is not or only poorly labeled, and another pool corresponding to the same triterpenes but located in the other plant cells and serving as phytosterol precursors, normally synthesized via the MVA pathway but with some contribution of the MEP pathway via the cross-talk between the cytosolic and the plastidial compartments (Scheme 6).

### 2.6. E. lathyris Isoprenoid Analysis by Natural Abundance IR GC-MS

Most plants are normally growing in phototrophic conditions, i.e., utilizing only CO_2_ as carbon source via photosynthesis. Feeding with an exogenous carbon source, even with a universal precursor like glucose, as this is done in labeling experiments, does not correspond to normal physiological conditions and may induce metabolic perturbations as compared to the metabolism under phototrophic growth. It is therefore tempting to approach metabolic pathways without performing feeding experiments. Enzyme reactions, like all chemical reactions, are submitted to isotope effects. Analyzing thus the isotope signature of metabolites may reveal features of their metabolic origin. The δ^13^C and the δ^2^H signatures are of peculiar value for analyzing metabolic pathways. MVA and MEP pathways correspond each to two completely different reaction sequences. Different isotope signatures are accordingly expected for isoprenoids issued from each of these pathways. This has been sometimes successfully verified, and the δ^13^C and the δ^2^H signatures allowed identifying the pathway involved in the biosynthesis of *Phaseolus lunatus* mono- and sesquiterpenoids [46,47]. In most cases, however the results were ambiguous and difficult to interpret [48,49,50,51]. We applied, however, this method to the isoprenoids of *E. lathyris* and of seven other plants and the unicellular alga *Scenedesmus obliquus* (data not shown). Results were by far not clear-cut and could not be interpreted in terms of MVA vs. MEP pathway for the origin of the isoprene units. They were only used qualitatively, pointing out a peculiar feature concerning hopenol B (**7**). All but one latex triterpenes as well as β-amyrin (**11**), which is not present in the latex but in other plant tissues, had similar depleted δ^2^H signatures between −200 and −243‰. Hopenol B (**7**), in contrast, with its −143‰ value, is completely outside the range of the former values. δ^13^C values of the triterpenes did not show such variations. Although hopenol B is synthesized via the mevalonate pathway like all other *E. lathyris* triterpenes, its odd δ^2^H signature indicates that it is issued from a different metabolic process.

## 3. Discussion and Conclusions

### 3.1. Latex Triterpenes are (Mainly) Synthesized via the MVA Pathway in E. lathyris

All incorporation experiments of labeled precursors showed that the isoprene units of all latex triterpenes from *E. lathyris* are solely synthesized via the MVA pathway, with no detectable contribution of the MEP pathway. From the incorporation of (1-^13^C)glucose and (1,6-^13^C_2_)glucose, only carbon atoms derived from C-2, C-4 and C-5 of IPP are labeled with a slight ^13^C enrichment at those deriving from C-1 and C-3 of IPP (Scheme 4, Table 3, Table 4 and Table 5). Carbon atoms derived from C-3 of IPP should be labeled neither by the MVA pathway, nor by the MEP pathway. The slight enrichment observed at this position most probably results from a scrambling occurring while ^13^C labeled glucose enters into several metabolic pathways resulting in a non-specific incorporation. The similar same scrambling is most probably true for the weak isotope abundance of the carbon atoms derived from C-1 of IPP, which are normally labeled in isoprene units issued from MEP pathway. The (^13^C)glucose incorporations are also in accordance with the dichotomy always observed in plants: sterols synthesized in the cytoplasm are issued from the MVA pathway whereas phytol is synthesized in the plastids and is derived from the MEP pathway [19,20,22,23]. Incorporations of (*3R*,*S*)-(2-^13^C)MVA and (5,5-^2^H_2_)DX are in accordance with this dichotomy observed in plants at the level of the cytoplasm and the plastids. The knowledge of the metabolic pathways utilized by *E. lathyris* for the biosynthesis of its latex triterpenes represents one of the first step required for the development of genetic transformation of the plant and to increase the triterpene content of its latex through genetic manipulation, improving *E. lathyris* as valuable isoprenoid factory. The fact that the MVA pathway solely contributes to the formation of the isoprene units of the latex clearly defines the main target for genetic transformation. The performed labeling experiments pointed, in addition, other interesting and rather unexpected features.

### 3.2. Compartmentation of Triterpenoid Biosynthesis and Possible Involvement of Endophytic Fungi for the Biosynthesis of Hopenol B

Labeling experiments pointed oddities in the isoprene unit biosynthesis in *E. lathyris*. Indeed, incorporation of (3*R*,*S*)-(2-^13^C)MVA at the highest concentration (1.9 mM) (and at a lesser extent at a lower 0.4 mM concentration of mevalonolactone) showed that labeled MVA was well incorporated into the sterols, which are synthesized in the cytoplasm of all cells and are essential constituents of the plasma membrane. Typical latex triterpenes such as lanosterol (**1**), butyrospermol (**5**) and hopenol B (**7**) with no known physiological role remained unlabeled, indicating that the MVA does not enter the laticifer compartment. Cycloartenol (**3**) and 24-methylene cycloartanol (**4**), however, distinguish themselves from the three former triterpenes (Table 6). The dual role of the cycloartane derivatives is most probably responsible for their differentiation leading to the presence of two non-interconvertible pools. On the one hand, cycloartenol (**3**) and 24-methylene cycloartanol (**4**) are easily labeled from MVA as cytosolic precursors of phytosterols in plant tissues outside the laticifers [52,53], whereas on the other hand the bulk of these two triterpenes found in laticifers cannot be labeled by MVA, much like lanosterol (**1**), butyrospermol (**5**) and hopenol B (**7**). The slight incorporation of (3*R*,*S*)-(2-^13^C)mevalonolactone into phytol (**12**) normally synthesized via the MEP pathway results from the cross-talk between cytosolic and plastidial compartments, which is quite often observed in plants [19,20,22,23].

The results of the incorporation of (5,5-^2^H_2_)DX were in accord with the conclusions reached after incorporation of (*3R*,*S*)-(2-^13^C)MVA. Deuterium labeled DX was very poorly incorporated into the *E*. *lathyri*s isoprenoids. ^2^H labeling could not be detected by conventional GC-MS. We had to switch to IR GC-MS for the characterization of labeled DX incorporation. Not surprisingly, phytol (**1**2) showed the highest deuterium enrichment (δ^2^H 1905‰). Sitosterol (**8**) was less significantly labeled (δ^2^H 472‰) corresponding to a major contribution of the MVA pathway with some minor contribution of the MEP pathway via the cross-talk. Lanosterol (**1**) and butyrospermol (**5**) were not labeled (δ^2^H respectively −114‰ and −94‰) indicating that the ^2^H labeled DX did not enter into the laticifers. Cycloartenol (**3**) and 24-methylene cycoartanol (**4**) (δ^2^H respectively 87‰ and 46‰) showed again an intermediate behavior, pointing again their dual origin as sterol precursors, which can be labeled to some extent by ^2^H labeled DX via the cytosolic/plastidial cross-talk and whose bulk pool in the laticifers remains unlabeled like lanosterol (**1**) and butyrospermol (**5**). Hopenol B (**7**) was in insufficient amounts to be analyzed by IR GC-MS.

A last quite intriguing aspect should be pointed out concerning hopenol B (**7**). This triterpene occupies a peculiar position amongst all latex triterpenes of *E. lathyris*. Upon incorporation of (1,6-^13^C_2_)glucose, hopenol B (**7**) showed a significantly higher ^13^C incorporation than those observed for other latex triterpenes (Table 5). More strikingly, the natural abundance δ^2^H signature of hopenol B (**7**) is completely outside the range of those of the other latex triterpenes (-143‰ vs. -200 and -243‰). These features strongly suggest that hopenol B (**7**) has a different metabolic origin in the laticifers than its four other companions. In addition, all genes corresponding to the oxidosqualene cyclases leading to cycloartenol (**3**), lanosterol (**1**) and butyrospermol (**5**) could be properly identified and the corresponding enzymes characterized [54]. Similar investigations, performed with the same successful methodology to find the gene responsible for a hopenol/oxidosqualene cyclase and the corresponding encoded enzyme failed [54]. As formation of butyrospermol (**5**) and hopenol B (**7**) results both from similar all-pre-chair folding of the acyclic oxidosqualene precursor [9,55], it was verified that the oxidosqualene/butyrospermol cyclase is not a versatile multi-product enzyme and produces only butyrospermol (**5**) as reaction product with no trace of hopenol B (**7**) [54]. It is therefore tempting to postulate that hopenol B (**7**) may not be synthesized by the plant in the laticifers but by an endophytic organism. Indeed, triterpenes of the hopane series are rather rare in higher plants [56], but quite common as 3-deoxy derivatives in bacteria [57] and in some ferns and mosses [56,58,59,60,61,62]. They are also present in lower fungi and in the fungal partners of lichens, either as 3-deoxytriterpenes (issued from squalene cyclization) or as 3β-hydroxy derivatives (issued from oxidosqualene cyclization) [56,63,64,65,66,67,68]. In fact, endophytic fungi are regularly found in plant tissues [69,70,71,72], including seeds [73,74,75,76,77] and latex [78]. In the latter case, they cannot be removed by the surface sterilization procedure we used to obtain the “axenic” plantlets, which still possessed in their tissues all endophytes previously present in the seeds.

Although the present work succeeded to show that latex triterpenes are derived from MVA pathway and showed the laticifer compartment was independent from the other plant tissues from the point of view of sterol, triterpene and phytol metabolism, it pointed out general problems of isoprenoid metabolism in plants highlighting the presence of pools of different metabolic origins. This difficult point deserves further investigations which cannot be easily investigated by labeling experiments or IR GC-MS, at least in their present state of development.

## 4. Materials and Methods

### 4.1. Plant Material

Seeds of *E. lathyris* were obtained from A. Boronat (University of Barcelona, Barcelona, Spain, [54]). Adult *E. lathyris* plants were grown outdoor in the garden of MR in Strasbourg. To get plantlets for incubation studies, seeds were surface sterilized by washing in 70% ethanol, rinsing with sterile water, soaking in 10% sodium hypochlorite for 5 min and final rinsing with sterile water. Germination was performed at 28 °C in the dark on filter paper soaked with a 1 mM GA3 solution (4 mL). When the rootlets were ca. 1–2 cm long, the plantlets were transferred in sterile 50 mL Falcon tubes containing Murashige-Skoog medium (Sigma-Aldrich, St-Quentin Fallavier, France) [31] containing 7% agarose. When the plantlets were about 2 cm high, they were transferred in sterile graduated 2 L glass cylinders containing the same solid culture medium and let to grow for 3 weeks on an Esat oriented window sill at room temperature and shaded daylight. For labeling experiments, (1-^13^C)glucose or (1,6-^13^C_2_)glucose (10 g.L^−1^, 99% isotope abundance, Omicron Biochemicals Inc., South Bend IN, USA), (3-*R*,*S*)-(2-^13^C)MVA (0.4 mM for mevalonolactone or 1.9 mM for mevalonate, 99% isotope abundance, Sigma-Aldrich, [23]) or (5,5-^2^H_2_)DX (98% isotope abundance, 1 mM, [23]) synthesized in the laboratory [41] were added during the whole culture. Plantlets (ca. 20–25, ca. 15 g, fresh weight) were harvested after ca. 3 weeks and freeze-dried. Labeled precursor and GA3 solutions were sterilized by filtration on Millex-GS (0.22 µ) filters (Merck-Millipore, Molsheim, France).

### 4.2. Analytical Methods

All commercial solvents (stored in glass bottles) were distilled to remove any trace of contaminants. Glassware was cleaned in a dish washer and thoroughly rinsed with a mixture of distilled chloroform and methanol (2:1, *v/v*) before use. Cotton utilized for filtrations was extracted for 48 h in a Soxhlet apparatus with the same chloroform and methanol mixture and air dried for one week.

Flash chromatography was performed on Si-60 silica gel (40–63 µm, Merck, Fontenay-sous-Bois, France) under nitrogen pressure [79].

For preparative TLC, Merck F_254_ glass plates (0.25 or 0.50 mm thickness) were used. For argentation chromatography, the plates were eluted with 15% AgNO_3_ (*w/v*) solution in acetonitrile, dried overnight in the dark at room temperature and activated at 110 °C for 1 h [80]. Compounds to be separated were applied with the minimum amount of cyclohexane. After elution, TLC plates were revealed using a non-destructive method by spraying with a 0.1% solution of berberine hydrochloride in 95% ethanol and by observing the plates under UV light (360 nm) [81]. Bands corresponding to the compounds of interest were scratched off, and the silica gel washed several times with dichloromethane.

^1^H- and ^13^C-NMR spectra were recorded at 298 K in C^2^HCl_3_ solution on Avance 300, Avance 400 or Avance 500 spectrometers (Bruker, Wissembourg, France). For amounts above 0.5 mg, standard NMR tubes (5 mm diameter), for smaller samples micro-tubes (2.5 mm diameter) were used. Chemical shifts are expressed in ppm using the CHCl_3_ signal for ^1^H-NMR (δ = 7.260 ppm) and the C^2^HCl_3_ signal (δ = 77.16 ppm) for ^13^C-NMR as references. For the incorporation of ^13^C labeled precursors, the isotope abundance of each carbon was determined as previously described [17,18,82,83] using as reference signal either that of a carbon atom corresponding to C-3 of IPP, which is not labeled neither by the MVA, nor by the MEP pathway, or those of the acetoxy methyl or carbonyl groups. Best results were obtained using the acetate methyl group as reference signal.

Gas chromatography was performed on an a 6850 apparatus (Agilent, Les Ulis, France) equipped with an on-column injector, a flame ionization detector and an Agilent HP5 capillary column (30 m × 0.32 mm, 0.25 μm film thickness) with dihydrogen as carrier gas. Injector and detector temperatures were respectively 55 °C and 290 °C. Steryl and triterpenyl acetates were injected in chloroform solution and analyzed with the following program: 3 min at 55 °C, 5 °C/min from 55 to 300 °C and 15 min at 300 °C.

GC-MS was performed on a Thermo TSQ Quantum spectrometer (Agilent, Les Ulis, France) coupled to Thermo Trace gas chromatograph. Source temperature was 220 °C. The spectrometer was utilized in positive electron impact mode. Compounds were injected in cyclohexane solution and analyzed on an Agilent HP5-MS column (30 m × 0.25 mm, 0.1 µm film thickness) with helium as carrier gas using the following program: 1 min at 80 °C, 3 °C/min from 80 to 310 °C and 5 min at 310 °C.

IR GC-MS was performed on a Thermo Delta V+ apparatus coupled to a Thermo Trace gas chromatograph equipped with an on-column injector and a high temperature combustion reactor set at 1000 °C for δ^13^C measurements and a pyrolysis reactor set at 1420 °C for δ^2^H measurements. GC separations were performed as described above for conventional GC-MS. Compounds were analyzed as trimethylsilyl ethers. Measured δ^2^H and δ^13^C values were corrected for the added trimethylsilyl group [84].

Cycloartenol (**3**) and 24-methylene cycloartanol (**4**) were available from our standard collection. Lanosterol (**1**) was purified from a commercial source. Butyrospermol (**5**) was extracted from fruits of *Maclura pomifera* [85] and hopenol B (7) was synthesized from 22-hydroxyhopanone as previously described [86].

### 4.3. Isolation of Triterpenes and Sterols

Freeze-dried plant material (ca. 2-3,5 g, plantlets) was shred with an Ultra Turrax T-25 (IKA-Werke, Staufen-im-Breisgau, Germany) blender in a minimum volume of a CHCl_3_/CH_3_OH mixture (2:1, *v/v*) and extracted under reflux for 1 h with the same solvent (3 × 50 mL). After evaporation to dryness of the combined extracts, the residue was saponified by refluxing for 1 h with Pa 6% KOH solution in methanol (3 mL/100 mg, *v/w*; KOH solution/extract). After addition of water (2 volumes), a first extraction with heptane (3 × 30 mL) afforded the non-saponifiable fraction. A second extraction after acidification with concentrated H_2_SO_4_ solution (pH = 2) and a similar heptane extraction afforded the crude fatty acid fraction. After drying the two extracts on anhydrous magnesium sulfate, they were evaporated to dryness.

The non-saponifiable fraction was separated by TLC on Merck silica gel glass plates (0.25 or 0.50 mm thickness) using dichloromethane as eluent (2 migrations) [81] and afforded two fractions that were recovered: a triterpene fraction containing also phytol, long chain fatty alcohols and triterpenols (R_F_ = 0.44) and a second fraction corresponding to sterols (R_F_ = 0.19).

Both fractions were acetylated overnight at room temperature with a mixture of acetic anhydride and pyridine (1:1, *v/v*, 300 µL). For the labeling experiments with an adult outdoor grown plant and the first axenic plantlets, acetylation was performed with (1,1′-^13^C_2_)-acetic anhydride (11% isotope abundance, Sigma-Aldrich) in order to amplify the signal of the acetate carbonyl group used as a reference signal for the determination of the isotope abundance of the labeled carbon atoms. As the isotope abundance was rather low, natural abundance acetic anhydride was used for all other acetylations. After evaporation of the excess of reagent under an argon stream, both acetates were separated by silica gel TLC (cyclohexane/ethyl acetate, 9:1). The fraction containing the triterpenol acetates was dissolved in a minimum of cyclohexane and further separated by TLC on silver nitrate impregnated silica gel (4 successive migrations cyclohexane/toluene, 7:3, *v/v*) yielding for a 180 mm migration, fatty alcohol acetates (125 mm), phytyl acetate (115 mm), lanosteryl acetate (90 mm), cycloartenyl acetate (80 mm), butyrospermyl acetate (60 mm) and a mixture of hopenyl B and 24-methylenecycloartanyl acetate (45 mm). All *E. lathyris* isoprenoids were identified by ^1^H- and ^13^C-NMR spectroscopy as well by GC-MS and comparison of the data with those of reference material.

Acetylation of alcohols is not suitable for IR GC-MS. The triterpenol fraction was dissolved in a minimum of cyclohexane and directly separated on silver nitrate impregnated silica gel plates (3 migrations, cyclohexane/absolute chloroform/acetone, 16:22:1). The resulting fractions were silylated with a mixture of *N*,*O*-bis(trimethylsilyl)trifluoroacetamide (BSTFA)/pyridine (4:1, *v/v*) at room temperature just before analysis by GC-MS or IR GC-MS. The δ^13^C and δ^2^H values of the BSTFA trimethylsilyl group were respectively −45.15‰ and −154.1‰.

## Figures and Tables

**Table 1:** Amounts of triterpenes and sterols isolated from *E. lathyris* samples.

			Amounts ^1^	
		Number of Analyses	Triterpene Fraction	Sterol Fraction
Whole plantlets from surface sterilized seeds		3	1.9	0.6
Whole plantlets grown in plant pots		2	1.5	0.8
Adult plants grown outdoor (>80 cm size)	aerial parts	3	7.5	1.0
	roots	3	traces, <0.1	0.7
	latex	4	20% ^2^	traces

^1^ mg·g^−1^, freeze-dried material. ^2^ *w/w*, freeze-dried latex.

**Table 2:** Average relative amounts of latex triterpenes (%) in *E. lathyris* samples.

	Plantlets ^1^	Adult Plants ^2^	Latex ^3^
Lanosterol **1**	18	18	25
Butyrospermol **5**	23	23	23
Cycloartenol **3**	17	16	18
24-Methylene cycloartanol **4**	29	29	29
Hopenol B **7**	7	9	6
Other triterpenes	5	5	3

^1^ Plantlets obtained from surface sterilized seeds and grow in sterile conditions and plantlets grown in plant pots on earth (four analyses). ^2^ Plants grown outdoor (four analyses). ^3^ Latex from plantlets mentioned above (four analyses).

**Table 3:** Average isotope abundances (%) of carbon atoms of *E. lathyris* isoprenoids from an adult plant grown in a plant pot after incorporation of (1-^13^C)glucose ^1^.

Isoprenoid	C-1	C-2	C-3	C-4	C-5	SAM ^2^
24-Methylene cycloartanol (**4**) ^3^	1.1	1.5	1.1	1.5	1.6	1.1
24-Methylene cycloartanol (**4**)	1.2	2.6	1.1	2.6	2.9	2.5
Cycloartenol (**3**)	1.3	3.6	1.1	3.7	3.7	-
Lanosterol (**1**)	1.3	3.5	1.3	3.4	3.4	-
Butyrospermol (**5**)	1.2	3.1	1.3	3.3	2.9	-
Hopenol B (**7**)	1.5	2.7	1.4	2.9	3.3	-
Sitosterol (**8**)	1.6	2.4	1.4	2.4	2.4	2.0
Phytol (**12**)	2.0	1.1	1.3	1.2	2.2	-

^1^ Mean values calculated for all equivalent carbon atoms from the biosynthetic point of view (IPP carbon atom numbering). Methyl signal of the acetoxy group was used as reference signal for the evaluation of the isotope abundances. ^2^ Carbon atoms derived from *S*-adenosyl methionine (SAM) by a methylation reaction. ^3^ 24-Methylene cycloartanol sample from a labeling experiment performed with an outdoor grown adult plant.

**Table 4:** Average isotope abundances (%) of carbon atoms of *E. lathyris* isoprenoids from plantlets obtained from surface sterilized seeds and grown in sterile conditions after incorporation of (1-^13^C)glucose ^1^.

	C-1	C-2	C-3	C-4	C-5	SAM ^2^
24-Methylene cycloartanol (**4**)	1.6	3.7	1.5	3.9	4.2	3.6
Cycloartenol (**3**)	1.8	4.9	1.7	5.3	5.2	-
Lanosterol (**1**)	1.4	4.5	1.6	4.8	4.7	-
Butyrospermol (**5**)	1.8	4.8	1.7	5.0	4.3	-
Hopenol B (**7**)	2.1	6.3	2.0	5.9	6.6	-
Sitosterol (**8**)	1.9	4.4	2.0	4.5	4.0	3.1
Phytol (**12**)	2.8	2.1	2.2	2.2	3.1	-

^1^ Mean values calculated for all equivalent carbon atoms from the biosynthetic point of view (IPP carbon atom numbering). Methyl signal of the acetoxy group used as reference signal for the evaluation of the isotope abundances. ^2^ Carbon atoms derived from *S*-adenosyl methionine (SAM) by a methylation reaction.

**Table 5:** Average isotope abundances (%) of carbon atoms of *E. lathyris* isoprenoids from plantlets obtained from surface sterilized seeds and grown in sterile conditions after incorporation of (1,6-^13^C_2_)glucose ^1^.

Isoprenoid	C-1	C-2	C-3	C-4	C-5	SAM ^2^
24-Methylene cycloartanol (**4**)	1.7	4.3	1.7	4.5	4.2	3.5
Cycloartenol (**3**)	1.9	5.5	1.8	5.7	6.0	-
Lanosterol (**1**)	1.8	5.0	1.8	5.2	5.5	-
Butyrospermol (**5**)	2.0	4.7	1.7	5.1	5.1	-
Hopenol B (**7**)	2.3	6.9	2.1	7.2	7.4	-
Sitosterol (**8**)	1.3	3.2	1.3	3.4	3.4	2.4
Phytol (**12**)	4.3	2.6	2.8	2.9	4.7	-

^1^ Mean values calculated for all equivalent carbon atoms from the biosynthetic point of view (IPP carbon atoms numbering). Methyl signal of the acetoxy group used as reference signal for the evaluation of the isotope abundances. ^2^ Carbon atoms derived from *S*-adenosyl methionine (SAM) by a methylation reaction.

**Table 6:** Average isotope abundances (%) of carbon atoms of *E. lathyris* isoprenoids from plantlets obtained from surface sterilized seeds and grown in sterile conditions after incorporation of (3*R*,*S*)-(2-^13^C)mevalonolactone (0.4 mM) or mevalonate (1.9 mM) ^1^.

	C-1	C-2	C-3	C-4	C-5	SAM ^2^
Incorporation of mevalonolactone						
24-Methylene cycloartanol (**4**)	1.1	1.2	1.1	1.6	1.2	1.4
Lanosterol (**1**)	1.1	1.1	1.1	1.0	1.1	-
Butyrospermol (**5**)	1.2	1.2	1.3	1.3	1.2	-
Sitosterol (**8**)	0.9	0.9	1.0	2.9	1.0	1.4
Phytol (**12**)	1.3	1.2	1.3	1.5	1.2	-
Incorporation of mevalonate						
24-Methylene cycloartanol (**4**)	1.1	1.2	1.1	4.3	1.2	1.3
Cycloartenol (**3**)	1.1	1.1	1.2	4.6	1.2	-
Lanosterol (**1**)	1.1	1.2	1.2	1.2	1.2	1.2
Butyrospermol (**5**)	1.1	1.1	1.1	1.1	1.3	-
Hopenol B (**7**)	1.0	1.1	1.3	1.3	1.3	-
Sitosterol (**8**)	1.0	1.1	1.1	5.7	1.1	1.0
Phytol (**12**)	1.1	1.1	1.1	1.4	1.1	-

^1^ Mean values calculated for all equivalent carbon atoms from the biosynthetic point of view (IPP carbon atom numbering). Methyl signal of the acetoxy group used as reference signal for the evaluation of the isotope abundances. ^2^ Carbon atoms derived from *S*-adenosyl methionine (SAM) by a methylation reaction.

## Abbreviations

GC	Gas chromatography
GC-MS	Gas chromatography coupled to mass spectrometry
IR GC-MS	Isotope ratio GC-MS
IPP	Isopentenyl diphosphate
MEP	Methylerythritol phosphate
MS medium	Murashige-Skoog medium
MVA	Mevalonate
NMR	Nuclear magnetic resonance
TLC	Thin layer chromatography
